# Pediatric Surgical Research Output in Germany in the Last 30 Years – An Assessment and International Comparison of Three Dedicated Paediatric Surgical Journals

**DOI:** 10.3389/fped.2020.00152

**Published:** 2020-04-22

**Authors:** Christina Oetzmann von Sochaczewski, Oliver J. Muensterer

**Affiliations:** Klinik und Poliklinik für Kinderchirurgie, Universitätsmedizin Mainz, Mainz, Germany

**Keywords:** bibliometrics, research productivity, geographical research distribution, institutional research distribution, academic surgery, citation analysis

## Abstract

**Purpose:** Research output of once-leading countries in surgical journals is decreasing despite an overall increase of scientific publications by 8% per year. We aimed to assess research outputs of German, Dutch, and Israeli pediatric surgeons in dedicated pediatric surgical journals in order to get insight into trends in pediatric surgical research.

**Methods:** We collected bibliographic information on all original articles in the *Journal of Pediatric Surgery, European Journal of Pediatric Surgery*, and *Pediatric Surgery International* in 1985–1988, 2000–2003, and 2015–2018 that had a German, Dutch or Israeli last author from a department of pediatric surgery. Citation counts were obtained from the Web of Science.

**Results:** Research output of German pediatric surgery decreased from 19 manuscripts in 1988 (0.1/surgeon/year) to eight manuscripts in 2017 (0.02/surgeon/year), whereas those of the Netherlands increased from two manuscripts in 1985 (0.08/surgeon/year) to 12 manuscripts in 2016 (0.3/surgeon/year). The declining German research output negatively correlated with increasing numbers of specialist pediatric surgeons for total (τ = −0.54; *P* = 0.0156) and manuscripts per surgeon (τ = −0.79; *P* = 0.0001), resulting in a negative trend over time (χ^2^ = 11.845, *P* = 0.0006). Analyses of citation patterns revealed that manuscripts by Dutch pediatric surgeons and those published in the *Journal of Pediatric Surgery* had higher absolute citation counts than the reference category of a German manuscript in the *European Journal of Pediatric Surgery*. Age-corrected citation rates resembled this result by increasing from 2000 to 2003 ($\tilde{\text{x}}$ = 0.799, range: 0–3.368) to 2015–2018 ($\tilde{\text{x}}$ = 2, range: 0–5) (*P* = 0.035) for the Netherlands. Assessment of manuscript types revealed that the proportion of prospective studies increased in the German sample (χ^2^ = 5.05, *P* = 0.0246), but remained the lowest among the comparators. Surprisingly, the proportion of non-clinical manuscripts from Germany also increased over time (χ^2^ = 4.001, *P* = 0.0455), whereas it remained constant in both the Netherlands and Israel.

**Conclusion:** German pediatric surgical research output decreased in the last thirty years based on the sample of dedicated pediatric surgical journals, while Dutch productivity increased. Citation rates—as a measure of scientific impact—were associated and increased with Dutch manuscripts. The involved factors remain to be determined and whether this represents a shift toward other journals or mirrors a general development.

## Introduction

The amount of scientific publications grew by 8% per year in the current decade (1), so did the number of articles published in three exclusively pediatric surgical journals, which has grown by 19% per decade in the last 30 years (2). More than twenty years ago, others noted that the share of formerly scientifically active countries in surgical journals decreased (3), despite the overall increase of journals and published research articles. This development has been accompanied by a steep reduction of surgical registrars that continue to participate in research activities after a temporary experience during training (4). We therefore aimed to assess whether the research output of German pediatric surgery within three dedicated pediatric surgical journals has also decreased and explored possible underlying factors that may have contributed to what some have called a “crisis of surgical research” (5). In order to add an international perspective, we compared the German situation to the Netherlands as a country that underwent intense centralization of pediatric surgical care (6), while it remained decentralized in Germany (7). Finally, we compared these results to Israel as a country whose structure of pediatric surgical care remained unchanged.

## Materials and Methods

We reviewed archives of three well-established journals solely devoted to pediatric surgery to assess the published research output of German pediatric surgery in a time span of 30 years. Based on the experience of others who only investigated 1 year per decade (2), we assessed 4 years in a row to account for random short-term variation in a representative sample. The most recent available year was 2018 and thus our analysis started in 1985. Based on the result that last authors are oftentimes responsible for conceptualization, planning, and supervising a study (8, 9), an item was counted if the last author was affiliated to a department of pediatric surgery, in line with the definition of previous bibliometric studies in the field of pediatric surgery (2). If not explicitly stated, this was confirmed assessing other publications with more or more detailed information on author affiliations, hospital websites or Researchgate profiles of the researcher in question. For international comparison, publication output of pediatric surgeons in the Netherlands and Israel was similarly assessed to determine absolute numbers of manuscripts published by pediatric surgeons. Only original articles—including systematic reviews and meta-analyses—were eligible for our study.

The mentioned countries were chosen, because we found reliable numbers of specialist pediatric surgeons within the literature: 55 pediatric surgeons from 17 departments from Israel in 1996 (10), 24 pediatric surgeons for the Netherlands at the Millennium (11), and 35 pediatric surgeons in 2017 (Table 1) (6).

**Table 1:** Number of hospital-based board-certified pediatric surgeons in the respective countries used to calculate the relative research output.

**Year**	**Netherlands**	**Germany**	**Israel**
1985	24	188	55
1986	24	180	55
1987	24	185	55
1988	24	184	55
1989		188	
1990		206	
1991		179	
1992		181	
1993		192	
1994		194	
1995		238	
1996		248	
1997		268	
1998		277	
1999		262	
2000	24	297	55
2001	24	318	55
2002	24	326	55
2003	24	319	55
2004		320	
2005		325	
2006		331	
2007		348	
2008		351	
2009		365	
2010		384	
2011		410	
2012		427	
2013		434	
2014		433	
2015	35	464	55
2016	35	492	55
2017	35	508	55
2018	35	521	55

The *Bundesärztekammer* (General Medical Council) publishes the number of all registered medical doctors in Germany each year including specialist pediatric surgeons working in hospitals (12). Pediatric surgeons exclusively treating outpatients (office-based), having solely administrative duties, and those being retired were excluded. The relative distributions among the sectors—hospital-based and outpatients treated in private practice—remained on similar levels throughout the study period. These data do not allow differentiating between pediatric surgeons working at academic or non-academic institutions. The relative percentage of specialist pediatric surgeons who worked in the former German Democratic Republic were estimated from their percentage of the total share in 1990 and added to numbers published by the *Bundesärztekammer* (General Medical Council) for the Federal Republic of Germany for 1985–1989 to obtain results for the pan-German territory. Absolute numbers of pediatric surgical manuscripts obtained from journal archives were divided by the number of specialist pediatric surgeons to obtain a relative research output parameter defined as the average of manuscripts per surgeon per year. A pediatric surgical center was defined as academic if it was affiliated with a medical school. In Germany, this number remained constant from 1985 to 2014, after which two additional schools were established, but none of the assessed manuscripts were from any of these two centers.

The number of specialist pediatric surgeons in Israel was assumed to be constant. Numbers of pediatric surgeons in the Netherlands in 1985–1988 and 2000–2003 were according to Ure and Bax (11), whereas the number of pediatric surgeons in 2015–2018 were those from Wijnen (6).

Citation data—presumed to be a measure of scientific impact (13)—for all included articles were obtained from Clarivate's Web of Science and normalized for publication date by division of the number of total cites with the years that have passed since publication up to 2019—yielding age-corrected citation rates—as described elsewhere to account for the power law distribution of citations (14).

We analyzed the relationship between the number of German specialist pediatric surgeons, relative research output, and absolute numbers of published manuscripts by Kendall's τ-b coefficient using R with its generic stats package (version 3.5.3) (15). The number of German specialist pediatric surgeons was analyzed using Pearsons's R and linear regression. The research output per board-certified surgeon, the proportion of clinically relevant and non-clinical manuscript types, and the number of manuscripts from non-academic centers were analyzed by the χ^2^-test for a linear trend. Differences between the pediatric surgical centers for a journal preference were assessed using the Fisher-Freeman-Halton test using the Bonferroni-correction for multiple comparisons for pairwise comparisons between the journals. Age-corrected citation rates were compared using the Kruskal-Wallis test followed by pairwise Wilcoxon signed-rank tests with a comparison for multiple tests using the Benjamini-Hochberg method (16). Absolute citation counts were analyzed by zero-inflated negative binomial regression using the R-packages pscl (version 1.5.2) for the zero-inflated negative binomial regression model (17), rcompanion (version 2.0.0) for the calculation of goodness-of-fit diagnostics (18), and emmeans (version 1.4.4) for groupwise comparisons of the regression results with *P*-value adjustments according to Tukey's method (19). In addition, goodness-of-fit was analyzed using the rootogram (20) implemented in the R-package countreg (version 0.2-1) (21).

We visualized distribution of manuscripts among German pediatric surgical centers in all analyzed timeframes using R's treemap-package (version 2.4-2) (22) in order to make them more accessible than larger data tables. Treemaps display data based on rectangle sizes proportional to a specified dimension (23), in our case the share of manuscripts of the total or of those in the three journals.

## Results

### The Quantitative Assessment: Number of Manuscripts and Numbers of Surgeons

**Figure 1:**
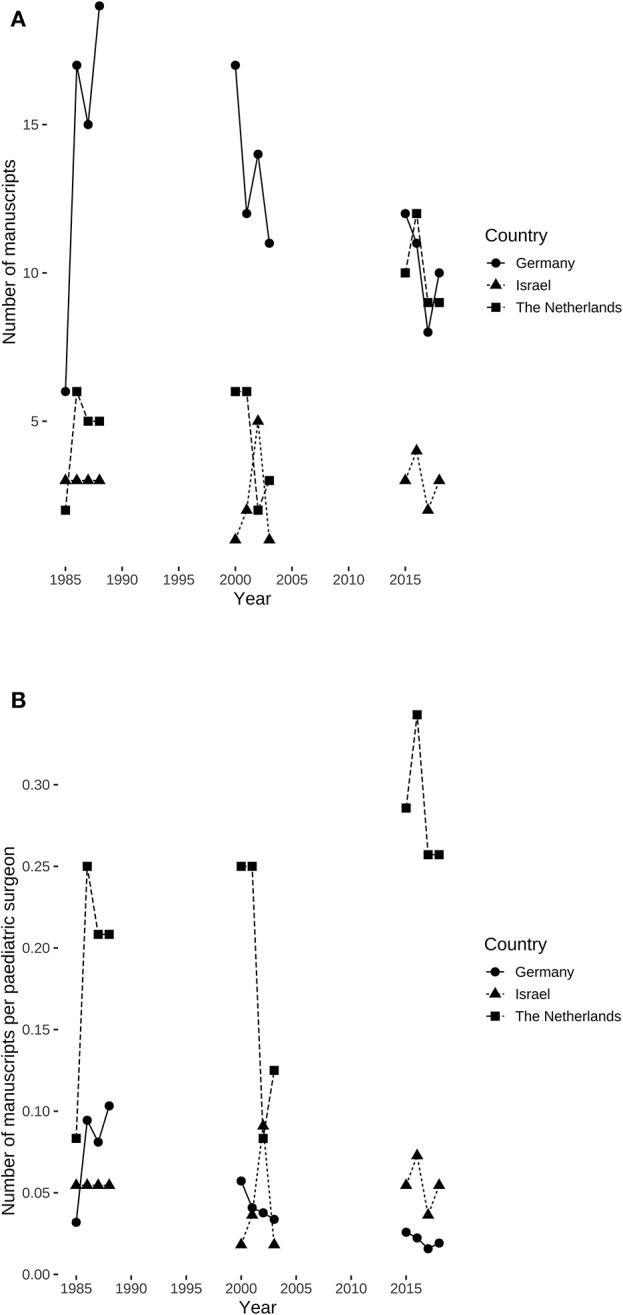
Absolute and relative number of manuscripts published in three pediatric surgical journals by examined countries. **(A)** Absolute number of manuscripts per country. **(B)** Relative research output in manuscripts per hospital-based pediatric surgeon specialist by country. Research output per surgeon declined over time (χ^2^ = 11.845, df = 1, *P* = 0.0006) in Germany, but neither in the Netherlands (χ^2^ = 0.607, df = 1, *P* = 0.436) nor in Israel (χ^2^ = 0, df = 1, *P* = 1).

Absolute numbers of manuscripts originating from Germany and published in the three selected pediatric surgical journals decreased over time from 57 in 1985–1988 to 41 in 2015–2018, whereas those from the Netherlands increased from 18 in 1985–1988 to 40 in 2015–2018. Meanwhile, those from Israel remained constant with 12 in 1985–1988 and 12 in 2015–2018 (Figure 1A). Similarly, the relative research output (manuscripts per board-certified hospital-based pediatric surgeons based on the numbers in Table 1) also experienced a decline in Germany from a mean 0.078 (standard deviation: 0.032) manuscripts per surgeon in 1985–1988 to a mean of 0.021 (standard deviation: 0.004) manuscripts per surgeon in 2015–2018. Contrary to this development, manuscripts per surgeon increased in the Netherlands from a mean of 0.188 (standard deviation: 0.072) in 1985–1988 to a mean of 0.286 (standard deviation: 0.04) manuscripts per surgeon, while it remained constant in Israel with 0.055 manuscripts per surgeon (Figure 1B).

**Figure 2:**
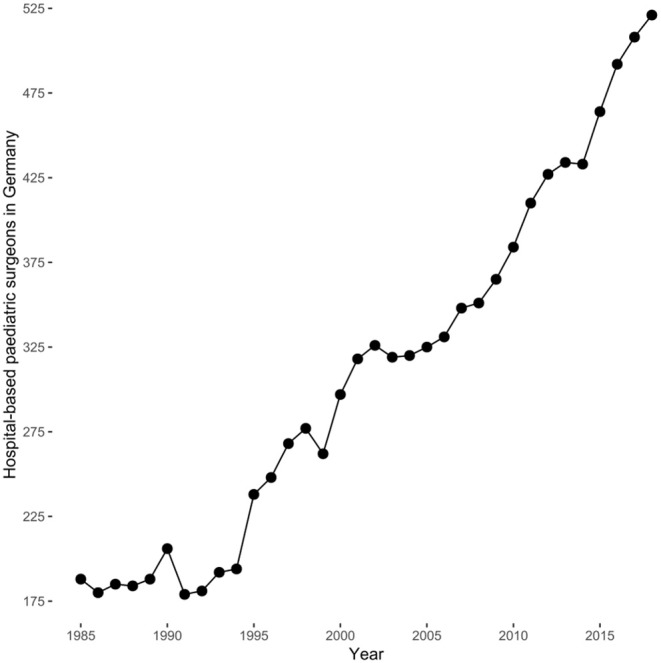
Hospital-based board-certified specialist pediatric surgeons in Germany by year according to the data published by the *Bundesärztekammer* (General Medical Council). They were highly correlated (*R* = 0.98, 95% confidence interval: 0.96–0.99, *P* < 0.0001) and showed a linear increase of 10.4 (95% confidence interval: 9.6–10.4) hospital-based board-certified pediatric surgeons per year (*F*_(1, 32)_ = 684.8, *P* < 0.0001).

**Figure 3:**
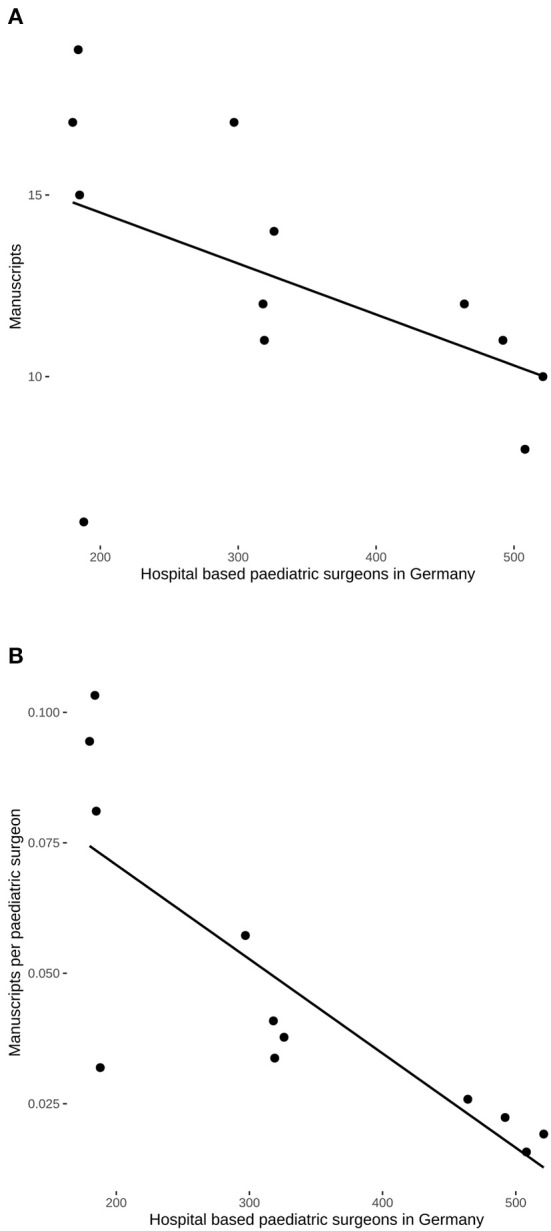
Correlation of hospital-based pediatric surgeons in Germany and scientific output in the three pediatric surgical journals. **(A)** The absolute number of manuscripts decreased with a concomitant increase in the number of hospital-based surgeons (τ = −0.54, *P* = 0.0156). **(B)** Highly negative correlation of the relative research output and the number of hospital-based pediatric surgeons (τ = −0.79, *P* = 0.0001).

The number of specialist pediatric surgeons in Germany increased steadily since the mid-1990s (Figure 2) being highly correlated (*R* = 0.98, 95% confidence interval: 0.96–0.99, *P* < 0.0001) and also showed a linear increase of 10.4 additional board-certified surgeons per year (*F*_(1, 32)_ = 648.8, 95% confidence interval: 9.6–11.2, *P* < 0.0001). Absolute output of published manuscripts negatively correlated with the number of hospital-based specialist pediatric surgeons in Germany (τ = −0.54, *P* = 0.0156) (Figure 3A). This negative relationship was also present for the relative research output in Germany with τ = −0.79 (*P* = 0.0001) (Figure 3B). Consequently, the research output per surgeon declined over time (χ^2^ = 11.845, df = 1, *P* = 0.0006) in Germany, but this was neither the case in the Netherlands (χ^2^ = 0.607, df = 1, *P* = 0.436) nor in Israel (χ^2^ = 0, df = 1, *P* = 1).

### Comparison of Journal Preferences by the Different Centers

**Figure 4:**
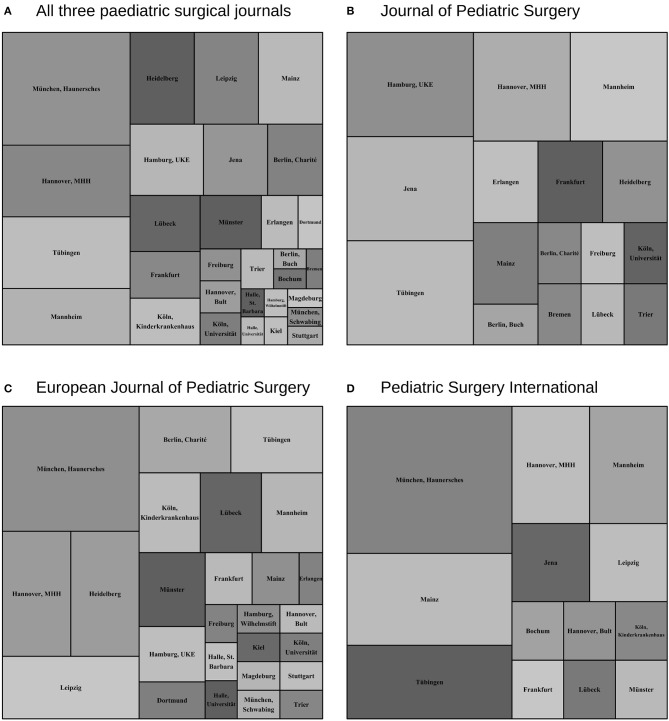
Treemap visualization of the cumulative share of manuscripts of the respective pediatric surgical center in the observed periods 1985–1988, 2000–2003, and 2015–2018. **(A)** Total manuscripts of all three journals. **(B)** Manuscripts in the *Journal of Pediatric Surgery*. **(C)** Manuscripts in the *European Journal of Pediatric Surgery*. **(D)** Manuscripts in *Pediatric Surgery International*. Preference of several pediatric surgical centers for some journals is indicated by their different representation among the sample (*P* = 0.0073), in which both the *European Journal of Pediatric Surgery* and *Pediatric Surgery International* differ from the Journal of Pediatric Surgery (*P* = 0.0063 and *P* = 0.0123, respectively), but not among each other (*P* = 0.414).

Analysis of total output revealed that pediatric surgical research in Germany is widely distributed among institutions (Figure 4A). It also reveals that some centers have preferences for publishing in certain pediatric surgical journals (*P* = 0.0073) (Figures 4B–D). Differences in journal preferences by different centers could be found in the comparison between the *Journal of Pediatric Surgery* and the *European Journal of Pediatric Surgery* (adjusted *P* = 0.0063) and between the *Journal of Pediatric Surgery* and *Pediatric Surgery International* (adjusted *P* = 0.0123), whereas there was no difference between the *European Journal of Pediatric Surgery* and *Pediatric Surgery International* (adjusted *P* = 0.414).

**Figure 5:**
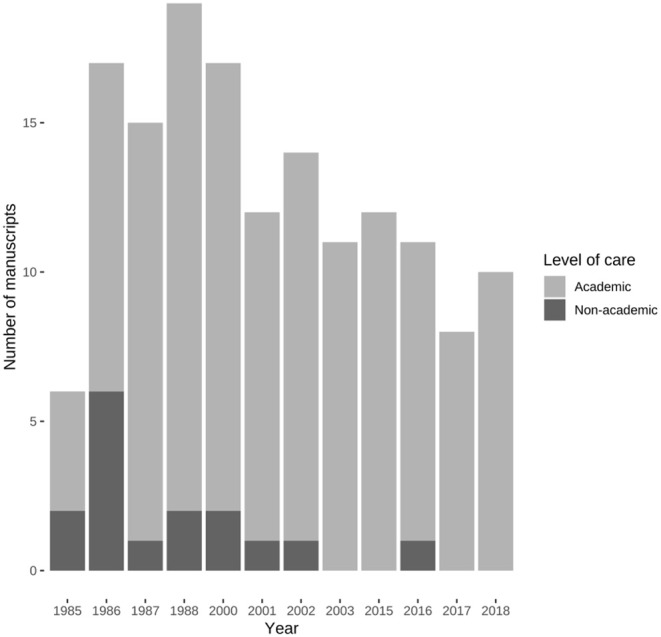
Manuscripts published by academic and non-academic pediatric surgical centers in Germany. The number of manuscripts from non-academic centers experienced a negative linear trend (χ^2^ = 10.9, df = 1, *P* = 0.001).

Our discrete time data revealed that once prolific scientific activities of many centers, non-academic ones in particular, had subsided exemplified by a decreasing linear trend for manuscripts from non-academic centers (χ^2^ = 10.9, df = 1, *P* = 0.001) (Figure 5).

### Comparison Between Age-Corrected Citation Data Between Countries

**Figure 6:**
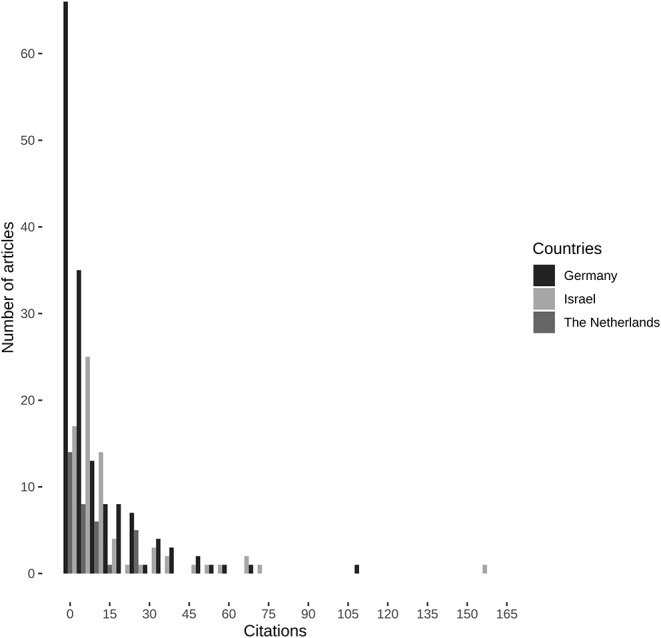
Absolute citation counts of all included manuscripts. Citation data obtained from Clarivate's Web of Science.

We compared the age-corrected citation data—obtained from the skewed absolute citations counts (Figure 6)—to test whether there were differences between the investigated countries: In the years 1985–1988, median age-corrected citations per manuscript differed (Kruskal-Wallis χ^2^ = 10.372, df = 2, *P* = 0.0056) between the Netherlands and Germany ($\tilde{\text{x}}$ = 0.226 vs. $\tilde{\text{x}}$ = 0.061, *P* = 0.0043), but not between the Netherlands and Israel ($\tilde{\text{x}}$ = 0.226 vs. $\tilde{\text{x}}$ = 0.118, *P* = 0.0761), and between Israel and Germany ($\tilde{\text{x}}$ = 0.061 vs. $\tilde{\text{x}}$ = 0.118, *P* = 0.573) (Table 2). In addition, there were no differences in age-corrected citation rates for both the years 2000–2003 (Kruskal-Wallis χ^2^ = 0.13, df = 2, *P* = 0.937) and 2015–2018 (Kruskal-Wallis χ^2^ = 5.398, df = 2, *P* = 0.0673) (Table 2).

**Table 2:** Age-corrected citation rates for the three compared countries in the three investigated timeframes in all three dedicated pediatric surgical journals.

**Years**	**Germany**	**Netherlands**	**Israel**
1985–1988	0.061 (0–1.677)	0.226 (0–4.727)	0.118 (0–0.788)
2000–2003	0.781 (0–5.684)	0.799 (0–3.368)	0.629 (0.056–1.688)
2015–2018	1 (0–4.5)	2 (0–5)	0.667 (0–6.75)

*Data are presented as median and ranges. Age-corrected citation rates are calculated from the absolute number of citations divided by the years since publication of an article up to 2019 as the latest year with complete citation counts. Statistically significant differences were present for Germany between 1985–1988 and both 2000–2003 (P <0.0001), and 2015–2018 (P <0.0001). For Israel, differences could be found between 1985–1988 and 2000–2003 (P = 0.015) and for the Netherlands between 1985–1988 and 2015–2018 (P = 0.011) and for the comparison of 2000–2003 with 2015–2018 (P = 0.035)*.

To test for differences in age-corrected citation rates over time, we compared the different investigated timeframes for each country. For Germany, the number of age-corrected citations differed (Kruskal-Wallis χ^2^ = 39.495, df = 2, *P* < 0.0001) between 1985–1988 and both 2000–2003 (*P* < 0.0001), and 2015–2018 (*P* < 0.0001), but not between the two later time periods (*P* = 0.51) (Table 2). A similar pattern could be observed for Israel as the differences in age-corrected citation rates (Kruskal-Wallis χ^2^ = 7.465, df = 2, *P* = 0.0239) differed between 1985–1988 and 2000–2003 (*P* = 0.015), but not to 2015–2018 (*P* = 0.081) and between both later timeframes (*P* = 0.637). For the Netherlands this pattern was different, because the increased age-corrected citation rates differed (Kruskal-Wallis χ^2^ = 11.141, df = 2, *P* = 0.0038), not between 1985–1988 and 2000–2003 (*P* = 0.215), but citation rates increased between 1985–1988 and 2015–2018 (*P* = 0.011). More importantly, age-corrected citation rates increased from 2000 to 2003 until the latest period between 2015 and 2018 (*P* = 0.035) (Table 2).

### Age-Corrected Citation Rates in the Three Dedicated Pediatric Surgical Journals

In order to test for differences between time periods in the different journals, we first evaluated them separately. It revealed that there were no differences for age-corrected citation rates in the *Journal of Pediatric Surgery* (Kruskal-Wallis χ^2^ = 5.244, df = 2, *P* = 0.0727) (Table 3), but differences were found for both the *European Journal of Pediatric Surgery* (Kruskal-Wallis χ^2^ = 33.435, df = 2, *P* < 0.0001) and *Pediatric Surgery International* (Kruskal-Wallis χ^2^ = 15.142, df = 2, *P* = 0.0005) (Table 3). These differences could be attributed to the smaller age-corrected citation rates in the period from 1985–1988 to 2000–2003 (EJPS: $\tilde{\text{x}}$ = 0.065 vs. $\tilde{\text{x}}$ = 0.947, *P* < 0.0001; PSI: $\tilde{\text{x}}$ = 0 vs. $\tilde{\text{x}}$ = 0.622, *P* = 0.0003) and 2015–2018 (EJPS: $\tilde{\text{x}}$ = 0.065 vs. $\tilde{\text{x}}$ = 1, *P* < 0.0001; PSI: $\tilde{\text{x}}$ = 0 vs. $\tilde{\text{x}}$ = 2.334, *P* = 0.0231), which did not differ when compared to each other (EJPS: $\tilde{\text{x}}$ = 0.947 vs. $\tilde{\text{x}}$ = 1, *P* = 0.62; PSI: $\tilde{\text{x}}$ = 0.662 vs. $\tilde{\text{x}}$ = 2.334, *P* = 0.1473) (Table 3).

**Table 3:** Age-corrected citation rates for the three compared dedicated pediatric surgical journals in the three investigated timeframes.

**Years**	**Journal of Pediatric Surgery**	**European Journal of Pediatric Surgery**	**Pediatric Surgery International**
1985–1988	0.375 (0–4.727)	0.065 (0–1.212)	0 (0–1)
2000–2003	1.158 (0–5.684)	0.947 (0–4.176)	0.622 (0–3.125)
2015–2018	2 (0–6.75)	1 (0–4)	2.334 (0–4.5)

*Data are presented as median and ranges. Age-corrected citation rates are calculated from the absolute number of citations divided by the years since publication of an article up to 2019 as the latest year with complete citation counts. Differences occurred in the European Journal of Pediatric Surgery for the comparison of 1985–1988 with both 2000–2003 (P <0.0001) and 2015–2018 (P <0.0001). These differences also occurred in Pediatric Surgery International (P = 0.0003 and P = 0.0231, respectively)*.

In addition, we conducted journal-wise comparisons to assess the relative contribution of the journals to the differences in age-corrected citation counts found in the assessment of overall citation data. For Germany, there were no differences in citation counts over time in the *Journal of Pediatric Surgery* (Kruskal-Wallis χ^2^ = 1.14, df = 2, *P* = 0.5656), but could be found in the *European Journal of Pediatric Surgery* (Kruskal-Wallis χ^2^ = 26.526, df = 2, *P* < 0.0001) and *Pediatric Surgery International* (Kruskal-Wallis χ^2^ = 8.31, df = 2, *P* = 0.0156) (Tables 4–6). Likewise, differences in age-corrected citation counts could not be found for both the Netherlands and Israel in the *Journal of Pediatric Surgery* (Kruskall-Wallis χ^2^ = 4.032, df = 2, *P* = 0.1332; Kruskal-Wallis χ^2^ = 3.887, df = 2, *P* = 0.1447, respectively), and *Pediatric Surgery International* (Kruskal-Wallis χ^2^ = 1.962, df = 2, *P* = 0.3749 and Kruskal-Wallis χ^2^ = 5.261, df = 2, *P* = 0.0721, respectively), (Tables 4, 6). In the *European Journal of Pediatric Surgery* differences in age-corrected citation rates were found for the Netherlands (Kruskal-Wallis χ^2^ = 6.877, df = 2, *P* = 0.0321), but not for Israel (Kruskal-Wallis χ^2^ = 0.7, df = 2, *P* = 0.7047) (Table 5).

**Table 4:** Age-corrected citation rates for the three compared countries in the three investigated timeframes in the Journal of Pediatric Surgery.

**Years**	**Germany**	**Netherlands**	**Israel**
1985–1988	0.369 (0–1.677)	0.758 (0–4.727)	0.246 (0–0.788)
2000–2003	1.135 (0–5.684)	1.095 (0–3.368)	1.353 (1.353–1.353)
2015–2018	1.5 (0–2.75)	2 (0–5)	1.834 (0.5–6.75)

*Data are presented as median and ranges. Age-corrected citation rates are calculated from the absolute number of citations divided by the years since publication of an article up to 2019 as the latest year with complete citation counts*.

**Table 5:** Age-corrected citation rates for the three compared countries in the three investigated timeframes in the European Journal of Pediatric Surgery.

**Years**	**Germany**	**Netherlands**	**Israel**
1985–1988	0.061 (0–1.212)	0.165 (0–0.912)	0.122 (0.059–0.242)
2000–2003	1 (0–4.176)	0.947 (0.5–2.579)	0.056 (0.056–0.056)
2015–2018	1 (0–4)	1.459 (0–3.667)	0.25 (0–3,75)

*Data are presented as median and ranges. Age-corrected citation rates are calculated from the absolute number of citations divided by the years since publication of an article up to 2019 as the latest year with complete citation counts. Statistically significant differences were found for Germany between 1985–1988 and both 2000–2003 (P = 0.0002) and 2015–2018 (P <0.0001)*.

**Table 6:** Age-corrected citation rates for the three compared countries in the three investigated timeframes in Pediatric Surgery International.

**Years**	**Germany**	**Netherlands**	**Israel**
1985–1988	0 (0–0.531)	0.162 (0.032–1)	0 (0–0)
2000–2003	0.5 (0–3.125)	0.722 (0.167–0.875)	0.629 (0.333–1.688)
2015–2018	3 (0–4.5)	2.25 (0–4.5)	2 (0–2.667)

*Data are presented as median and ranges. Age-corrected citation rates are calculated from the absolute number of citations divided by the years since publication of an article up to 2019 as the latest year with complete citation counts. Differences could be found for Germany between 1985–1988 and 2000–2003 (P = 0.01)*.

Assessment of the differences for the Netherlands revealed that the difference in the Kruskal-Wallis omnibus-test vanished in the groupwise-comparison of age-corrected citation rates in the *European Journal of Pediatric Surgery* for all investigated timeframes (1985–1988 vs. 2000–2003: $\tilde{\text{x}}$ = 0.165 vs. $\tilde{\text{x}}$ = 0.947, *P* = 0.095; 1985–1988 vs. 2015–2018: $\tilde{\text{x}}$ = 0.165 vs. $\tilde{\text{x}}$ = 1.459, *P* = 0.055; 2000–2003 vs. 2015–2018: $\tilde{\text{x}}$ = 0.947 vs. $\tilde{\text{x}}$ = 1.459, *P* = 0.528) (Table 5). For Germany, differences stemmed from much lower citation counts in 1985–1988 compared to 2000–2003, and 2015–2018 for the *European Journal of Pediatric Surgery* ($\tilde{\text{x}}$ = 0.061 vs. $\tilde{\text{x}}$ = 1, *P* = 0.0002 and $\tilde{\text{x}}$ = 0.061 vs. $\tilde{\text{x}}$ = 1, *P* < 0.0001, respectively), and for 1985–1988 compared to 2000–2003 in *Pediatric Surgery International* ($\tilde{\text{x}}$ = 0 vs. $\tilde{\text{x}}$ = 0.5, *P* = 0.01) (Tables 5, 6). In contrast, there were no differences in age-corrected citation counts for the comparisons of 2000–2003 with 2015–2018 in both the *European Journal of Pediatric Surgery* and *Pediatric Surgery International* ($\tilde{\text{x}}$ = 0.947 vs. $\tilde{\text{x}}$ = 1.459, *P* = 0.988 and $\tilde{\text{x}}$ = 0.5 vs. $\tilde{\text{x}}$ = 3, *P* = 0.52, respectively), (Tables 5, 6). In the latter, the age-corrected citation counts were also similar between 1985–1988 and 2015–2018 ($\tilde{\text{x}}$ = 0 vs. $\tilde{\text{x}}$ = 3, *P* = 0.28) (Table 6).

### Regression Modeling of Absolute Citation Counts

**Figure 7:**
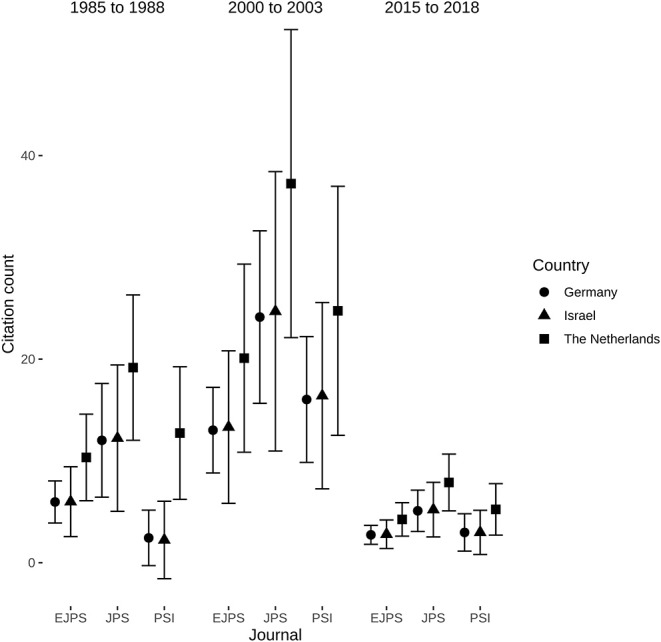
Journal- and country-wise comparisons of absolute citation counts in the three investigated timeframes. Data represents mean and 95% confidence intervals calculated from the zero-inflated negative binomial regression presented in Table 7. Non-overlapping confidence intervals indicate significant differences. *P*-values were corrected for multiple comparisons using Tukey's method.

We then used regression-analysis to assess whether there would be differences in rates of absolute citations among the comparators and journals. Zero-inflated negative binomial regression (Nagelkerke's pseudo-*R*^2^ = 0.301, Likelihood-Ratio-Test: log likelihood difference = −47.55, *P* < 0.0001) revealed that compared to the intercept for a German manuscript in the *European Journal of Pediatric Surgery* in 1985–1988, a manuscript published in the *Journal of Pediatric Surgery*, a manuscript published between 2000 and 2003, and a manuscript published by Dutch pediatric surgeons had higher rates of absolute citation counts, whereas publication between 2015 and 2018 indicated lower absolute citation counts (Table 7). Consequently, manuscripts by Dutch pediatric surgeons had higher absolute citations counts as had those that were published in the *Journal of Pediatric Surgery*, whereas newer manuscripts were penalized for the low amount of time that has passed since publication and thus precluding accumulation of citations as their absolute number of citations did not exceed those manuscripts published in the founding years of *Pediatric Surgery International* or those manuscripts that were mainly written in German in the *Zeitschrift für Kinderchirurgie* (Figure 7).

**Table 7:** Zero-inflated negative binomial regression of absolute citation counts as shown in Figure 6.

**Predictor**	**Estimate**	**Standard error**	**Z-Score**	***P*-value**
Reference	1.902	0.186	10.242	<0.0001
Journal of Pediatric Surgery	0.617	0.179	3.445	0.0006
Pediatric Surgery International	0.208	0.218	0.956	0.3391
Israel	0.023	0.253	0.091	0.9273
Netherlands	0.434	0.186	2.328	0.0199
2000–2003	0.664	0.205	3.239	0.0012
2015–2018	−0.889	0.197	−4.513	<0.0001
Log (θ)	−0.226	0.113	−2.002	0.0453

*Analyses of Goodness-of-fit: Nagelkerke's pseudo-R^2^ = 0.308 (Likelihood-ratio test: Log likelihood difference: −47.55, P <0.0001). Akaike information criterion = 1637.788. Bayesian information criterion = 1691.14. Number of iterations in Broyden-Fletcher-Goldfarb-Shanno (BFGS) optimization: 63 and log likelihood = −803.9 with 15 degrees of freedom. θ represents the dispersion parameter used to calculate the variance together with the mean and therefore represents the difference to the Poisson-distribution in which mean and variance have to be equal*.

### Analysis of Scientific Quality by Manuscript Type

As citation counts may not be a suitable indicator of scientific quality, we compared the different manuscript types over time (Table 8). The proportion of prospective studies experienced a linear increase in manuscripts from Germany (χ^2^ = 5.05, df = 1, *P* = 0.0246), but not in those from the Netherlands (χ^2^ = 1.019, df = 1, *P* = 0.3128) or Israel (χ =0.695, df = 1, *P* = 0.4046), although the proportion of prospective manuscripts overall is the lowest in manuscripts from Germany (Table 8). It is often argued that basic science manuscripts would be submitted to more prestigious journals, one would expect the proportion of non-clinical manuscripts to decrease over time. We also tested this by assessing their proportion for a linear trend and found that the proportion of non-clinical manuscripts in the three dedicated pediatric surgical journals increased for manuscripts from Germany (χ^2^ = 4.001, df = 1, *P* = 0.0455), whereas there was no such trend for Dutch manuscripts (χ^2^ = 0.909, df = 2, *P* = 0.3403) or those from Israel (χ^2^ = 1.059, df = 1, *P* = 0.3035) (Table 8).

**Table 8:** Proportion of manuscript types by country and Journal in the investigated timeframes.

	**Germany**			**Netherlands**			**Israel**		
	**Clinical**		**Non-clinical**	**Clinical**		**Non-clinical**	**Clinical**		**Non-clinical**
	**Retro-spective**	**Pro-spective**		**Retro-spective**	**Pro-spective**		**Retro-spective**	**Pro-spective**	
1985–1988	0.898	0	0.102	0.579	0.105	0.316	0.923	0	0.077
2000–2003	0.618	0.018	0.364	0.667	0.417	0.06	0.625	0.125	0.25
2015–2018	0.638	0.085	0.277	0.769	0.063	0.18	0.692	0.077	0.231

*Manuscript types presented as proportions to account for differences in absolute numbers. The proportion of non-clinical manuscripts followed an increasing linear trend for Germany (χ^2^ = 4.005, df = 1, P = 0.0455) as did the proportion of prospectively conducted studies (χ^2^ = 5.05, df = 1, P = 0.0246)*.

## Discussion

We found a decreasing amount of original articles published by German departments of pediatric surgery in three leading pediatric surgical journals over the last three decades.

### A Similar Situation in General Surgery

Declining numbers of original articles in our study fit well with storylines of what others described “endangered” (24) or “extinct” (5) surgeon-scientists. Similar results of decreasing research productivity in surgical research journals were not only described for general surgery in the United States (3), but also for German general surgery based on *Langenbeck's Archive of Surgery* (25) as well as for the United Kingdom and Ireland (26). Increasing methodological demands cannot be met by research activities following a regular work day, which is still common despite all pleas against it (25, 27–29) and does not exclude pediatric surgery (30). It therefore represents an inexpungable obstacle for non-academic surgeons in Germany as non-clinical duties are neither financially compensated nor perceived part of the contract and thus scientific work takes place during spare time and off-hours (27, 28). In German general surgery, the vast majority of operations are conducted at non-academic departments that do not participate in research (31). Thus, a network was started to initiate and coordinate surgical research in Germany to overcome this development (31) by providing services to ease research activities and to help in knowledge transfer to non-academic centers (32). The efficacy of this network has been evaluated by comparing participating to non-participating hospitals and found that participating hospitals have more personal formally qualified in conducting clinical research, more often qualify their doctors to become a clinical investigator, participate in more multicenter trials, and have more supporting infrastructure (33), thus improving scientific quality and quantity of surgical research in Germany (32, 33).

### The International Perspective: The Netherlands

Increasing productivity of the Dutch surgeons has been noted in other disciplines too and may be linked to major differences in department structure and perception on the necessity of research besides clinical duties (28, 34, 35). It has been suggested that a relevant factor may be the smaller number of surgeons accompanied by better cooperation compared to larger countries with a higher surgeon-workforce (35). In the Netherlands, it was in the 1960s when pediatric surgeons coined the separation of surgery in children, which could be conducted by general surgeons too, and specialized pediatric surgery exclusively performed by pediatric surgeons (11). In 1989, specialized pediatric surgical care was clearly defined by the Dutch ministry of health and accompanied by a concentration of pediatric surgical care in just six centers chosen by the Dutch association of pediatric surgery (11). Further contributing factors for specialization were restriction of neonatal intensive care to only eight centers and referral of selected age groups to specialized anesthesiology care (11). A recent development was a further centralization that requires centers to treat at least ten patients per year and condition, again introduced by the Dutch association of pediatric surgeons (6). Moreover, a mandatory database was introduced in order to check the performance of individual centers, which currently is not a research tool, but enforces strict documentation of a core dataset (6). In line with others (35), we speculate that these developments have promoted academic productivity in pediatric surgery, too.

Contrary to Germany, pediatric surgery is not recognized as a separate specialty in the Netherlands, but performed by a small group of general surgeons specifically trained for this task after finishing their postgraduate education in general surgery (11). Much to our surprise, we found that the Netherlands were more scientifically active and productive than Germany. Moreover, their scientific impact based on the number of absolute and age-corrected citation counts increased in the last two decades, whereas it remained unchanged for Israel and Germany. This result may indicate that the access to a general, interdisciplinary infrastructure may be more important—and ultimately more promising—than relying on resources within pediatric surgery alone.

In contrast, German pediatric surgery follows a decentralized approach (7). The number of departments has been relatively constant in the period of investigation. Schmedding and Rolle reported 89 departments in 1984 for East and West Germany (7), which decreased to 83 departments with 2,853 hospital beds in 1997, 76 departments with 1,920 beds in 2007, and 90 departments with 1,740 hospital beds in 2017 according to the *Statistisches Jahrbuch der Bundesrepublik Deutschland* (annual report of the German statistics agency). Contrary to the Netherlands, there currently is no definition of specialized pediatric surgical care or designation of referral centers for certain pediatric surgical diseases by the *Deutsche Gesellschaft für Kinderchirurgie* (German association of pediatric surgery) or by the respective legislative bodies. Therefore, more and more specialist pediatric surgeons serve a shrinking number of children in Germany who occupy fewer hospital beds than 20 years before. A development accompanied by a decreasing research output without a relevant improvement in scientific quality based on the proportion of prospective studies, which increased, but remained lower than in the sample of the comparators. Although one would expect an increase of research productivity as a higher workforce on a smaller number of patients suggests an increased availability of time for research activities. As this seems not be the case, the underlying causative factors need to be explored further.

### Causative Factors for a Declining Research Output in Surgery

A postulated major factor for a reduced research output has been a decrease in external funding for surgical research (4, 36), which has been identified as a highly relevant factor for a successful scientific career in academic surgery (37). An increasing competition and thus a low success rate for variable funding with decreasing fixed funding has been described for Germany and the Netherlands (38–40). Likewise, the success rate for grants has also experienced a decline over time in Israel (41). Besides this financial factor, excessive burden of time due to clinical duties for junior doctors (4, 28, 36, 42) and administrative tasks for their senior counterparts (4, 34) were identified by others as barriers to successful research.

In addition, a lack of formal research training has repeatedly been stated to highly contribute toward unsuccessful and inefficient research experiences by surgical registrars in the United States (36), South Africa (43), Europe (44), and Germany (28) in particular. This might be a factor in the observation of almost halved number of basic science abstracts presented at the Academic Surgical Congress (4). An observation that has also been made at pediatric surgical meetings, although in a less pronounced manner, particularly at the expense of basic science abstracts (45). Although speculative, it is in turn likely that a lack of formal research training is an important contributing factor in declining research outputs: Compared to a non-compulsory series of research lectures, structured research training within the first years of surgical training resulted in a doubled research output of published manuscripts, which also were of a higher scientific quality (46). This is supported by the finding that success of a student research project is determined by available guidance in methodology, whereas failure is closely linked to a lack thereof (47).

Importance of mentorship for successful surgical research is stressed universally: Either in its positive form as a contributing factor for success (36, 37, 42, 44) or in its negative form due to a lack of mentorship resulting in failed research projects (43, 47–49).

### Proposed Solutions: Formal Research Training and Dedicated Research Staff

Consequently, a roadmap has been developed that takes all these aspects into account to outline necessary steps to achieve during the long and windy road toward a successful surgeon-scientist (50). Importance of both mentorship and methodological excellence may be exemplified by the superior performance of MD/PhDs compared to MDs in scientific output and extramural funding (51). Moreover, PhDs working as dedicated research staff at surgical departments exhibit a relevant pull effect on MDs at the same department: They not only become more likely to obtain external funding, but their scientific output is comparative to colleagues working at highly funded departments (51). This suggests that dedicated research staff may improve research in particular at lower resource departments (51).

### Citation Data

The number of age-corrected citation counts increased, mainly between the first timeframe from 1985 to 1988 compared to the later ones, whereas higher age-corrected citations counts between 2000–2003 and 2015–2018 could only be found for Dutch pediatric surgeons. This result was in line with the regression analysis of absolute citation counts that were higher in manuscripts from the Netherlands and those published in the *Journal of Pediatric Surgery*. Differences in the earlier timeframe is likely to be attributed to *Pediatric Surgery International* having just been founded, which was a major drawback when paper copies were not widely circulated due to being new and electronic information systems were still to be constructed. The majority of articles in the predecessor of the *European Journal of Pediatric Surgery*—the *Zeitschrift für Kinderchirurgie*—were largely written in German, which inevitably reduces citation rates as non-English literature is cited less often (52–54). Although our analysis is likely to be more representative than previous works that used a single year to extrapolate a decade (2), it could be the case that the non-investigated years might change the results as a more complete assessment might enable regression analysis of age-corrected citation counts, which currently is impossible as the distribution could not be adequately modeled by available distributions. This should however be conducted by including the expertise of colleagues with particular experience in scientometrics.

### Limitations of Our Study

A relevant limitation is the restriction to three leading pediatric surgical journals. Since some centers are highly specialized and may therefore focus on publications in journals outside the pediatric surgery core journals. Not including these specialized journals precludes comparative evaluation of departments. However, our analysis was never intended as an interdepartmental comparison, but as a global view of the big picture on German academic pediatric surgical output.

More importantly, we felt a relevant trend might solely be observed using data from several decades, which only long-standing established society journals (55) such as those devoted to pediatric surgery may provide. It may however be argued that our study design does not cover more important journals based on their journal impact factor ranking, because high-quality surgical research may have been published in them (25). Apart from the fact that Eugene Garfield—the inventor of the journal impact factor—never intended his invention to be used to evaluate individuals (56), it gained its importance due to the widespread use as a tool to not only evaluate and compare the scientific work of persons, but by its dominance in allocating intramural funds (57). Publishing decision solely based on journal impact factor rankings may have devastating consequences if a journal suddenly gets delisted by the Institute of Scientific Information: Exemplified by the once thriving journal *Oncotarget*, which was excluded in January 2018 resulting in many manuscripts losing their intended value as its journal impact factor is zero afterwards (58).

Limitations of the journal impact factor have often been discussed (59), but previous reports have shown that basic science manuscripts are more prone to be published outside the surgical literature than clinical manuscripts important to the field (25). Consequently, pediatric surgical departments with a strong focus on basic research may be underrepresented by focusing on the selected journals, because they may choose to publish their basic science manuscripts elsewhere. As such, one would have expected the proportion of non-clinical manuscripts published the in three dedicated pediatric surgical journals to decrease. However, their proportion in the total share of German pediatric surgical research output even increased in the investigated timeframes, which is counterintuitively to the point that basic research might be published in more specialized journals. Nevertheless, the three dedicated pediatric surgical journals represent the most circulated and read publications in the cross-sectional field of pediatric surgery. Therefore, our assessment of the three journals devoted to pediatric surgery is likely to provide a useful insight into scientific activity of pediatric surgery.

Another limitation of our study is the much less detailed information on numbers of pediatric surgeons in other countries, which could only be obtained from the literature and thus reduce comparability of relative numbers of scientific output. However, exact data seem not to be available in other countries, which may be highlighted by an author from the Netherlands describing “[…] **approximately 35** certified pediatric surgeons,” despite the pediatric surgical community being small compared to other countries (6). Nevertheless, exact numbers of time a required to conduct reliable comparisons and might only be obtained by a multi-centric approach of pediatric surgeons based in the respective countries who are familiar with their system and community.

## Conclusion

Scientific output of German pediatric surgery in dedicated pediatric surgical journals has decreased in both absolute and relative numbers in the last 30 years caused by a concentration of research activities at some prolific, mostly academic, centers. On the contrary, the contribution of Dutch pediatric surgeons to dedicated pediatric surgical journals has increased over time and has been associated with increased numbers of both absolute and age-corrected citation counts as a measure of scientific impact. Our present study provides a first insight into research productivity of three countries in selected timeframes, but reliable answers whether this is just a snap-short or mirroring a general development needs to be assessed taking wider timeframes into account and preferably more countries in a multi-centric approach in order to account for different system architectures and cultures within them.

## Data Availability Statement

All data analyzed for this study are included in the article.

## Author Contributions

CO and OM designed the study. CO collected the data and analyzed them. OM made random contingency checks of data collection. CO wrote the first draft, which was revised by OM. Both authors read and approved the final submission.

## Conflict of Interest

The authors declare that the research was conducted in the absence of any commercial or financial relationships that could be construed as a potential conflict of interest.
